# Supplementary material for: mTORC1 in AGRP neurons integrates exteroceptive and interoceptive food-related cues in the modulation of adaptive energy expenditure in mice
Source: eLife. 2017 May 23;6:e22848. doi: 10.7554/eLife.22848 (PMC5441868; doi:10.7554/eLife.22848)
Supplement: Supplementary file 1. — DOI: http://dx.doi.org/10.7554/eLife.22848.014 [file elife-22848-supp1.docx]

**Supplementary Table 1. Real time PCR Oligonucleotide Sequences**

| Target Gene | Forward Primer | Reverse Primer | Probe |
| --- | --- | --- | --- |
| Mouse pGC1a | AAC CAC ACC CAC AGG ATC AGA | CTC TTC GCT TTA TTG CTC CAT GA | CAA ACC CTG CCA TTG TTA AGA CCG AGA A |
| Mouse Elovl6 | TGC AGG AAA ACT GGA AGA AGT CT | ATG CCG ACC ACC AAA GAT AAA |  |
| Mouse 18S | CGG CTA CCA CAT CCA AGG AA | GCT GGA ATT ACC GCG GCT |  |
| Mouse 36b4 | AGA TGC AGC AGA TCC GCA T | GTT CTT GCC CAT CAG CAC C |  |
| Mouse βactin | GCT CTG GCT CCT AGC ACC AT | GCC ACC GAT CCA CAC AGA GT | ATC AAG ATC ATT GCT CCT CCT GAG CGC |
| Mouse mUCP1 | CCC GCT GGA CAC TGC C | ACC TAA TGG TAC TGG AAG CCT GG | AAG TCC GCC TTC AGA TCC AAG GTG AAG |
| Mouse mD2 | TGC GCT GTG TCT GGA ACA G | CTG GAA TTG GGA GCA TCT TCA |  |
